# Triglyceride-glucose index and stroke recurrence in elderly patients with ischemic stroke

**DOI:** 10.3389/fendo.2022.1005614

**Published:** 2022-08-29

**Authors:** Fang Wang, Jinjing Wang, Yunfei Han, Xuan Shi, Xiaohui Xu, Chao Hou, Jie Gao, Shuanggen Zhu, Xinfeng Liu

**Affiliations:** ^1^ Department of Neurology, Affiliated Jinling Hospital, Medical School of Nanjing University, Nanjing, China; ^2^ Department of Neurology, The Affiliated Central Hospital of Shenzhen Longhua District, Guangdong Medical University, Shenzhen, China; ^3^ Department of Neurology, People’s Hospital of Longhua, Shenzhen, China

**Keywords:** TyG index, ischemic, stroke, elderly, recurrence

## Abstract

**Background and purpose:**

This study aimed to explore the association between triglyceride–glucose (TyG) index and stroke recurrence in elderly patients with ischemic stroke in China.

**Methods:**

We enrolled ischemic stroke patients aged ≥ 65 years from the Nanjing Stroke Registry Program. The primary endpoint was defined as recurrent stroke within one year after the index stroke. We used multivariable Cox proportional hazards regression models to investigate the association between TyG index and stroke recurrence. We assessed the discriminative ability of TyG index with the receiver operative characteristic and the area under the curve.

**Results:**

A total of 955 patients (median age, 70.0 [67.0, 75.0]; male sex, 67.2%) from the Nanjing Stroke Registry Program were enrolled. During one year follow-up, 97 (10.2%) elderly patients experienced stroke recurrence. In multivariable analyses, the association between TyG index and stroke recurrence remained significant after adjusting for confounders (quartile 4 versus quartile 1; hazard ratio, 2.073, 95% confidence interval, 1.158-3.711; *P* = 0.014). The restricted cubic spline showed an increasing trend for TyG index and stroke recurrence (*P* for non-linearity = 0.072). The area under the curve to predict stroke recurrence with TyG index was 0.719 (95% confidence interval, 0.666-0.772). Besides, TyG index slightly improved the prediction for stroke recurrence.

**Conclusion:**

Elevated TyG index was associated with stroke recurrence in elderly patients with ischemic stroke. Further studies are warranted to assess the role of TyG index in the development of stroke recurrence in the elderly.

## Introduction

Stroke is a leading course of mortality and disability worldwide (1). Due to multiple risk factors, ischemic stroke increases with advancing age and is associated with poor prognosis in elderly patients (2). Prior studies reported that over 75% of strokes occurred in the elderly and added a heavy economic burden (3). Furthermore, elderly patients have an increased risk of vascular events compared with younger adults (4). With a life expectancy of a least five years ahead, it is warranted to identify elderly patients with higher risk of stroke recurrence (5).

Insulin resistance (IR) is a pathological state caused by increased insulin sensitivity and the precursor of diabetes mellitus (6). Previous studies revealed that IR promotes stroke progression and is associated with poor prognoses (7–9). The golden standard measurement for IR, hyperinsulinemic-euglycemic clamp, is not commonly used in clinical practice due to the cost and complexity (10). Notably, triglyceride–glucose (TyG) index, which is derived from fasting blood glucose and triglyceride, is a convenient surrogate marker of IR (11). Prior studies suggested that TyG index is associated with nonalcoholic fatty liver disease (12), acute coronary syndrome (13), and hyperuricemia (14) in elderly patients. However, few studies have investigated the association between TyG index and the risk of stroke recurrence in elderly patients with ischemic stroke.

Hence, we conducted this study to explore the potential role of TyG index in elderly patients with ischemic stroke.

## Methods

The data that support the findings of this study are available from the corresponding author on reasonable request.

### Participants

From January 1 2013 to October 31, 2016, patients with ischemic stroke were continuously enrolled from the Nanjing Stroke Registry Program (15). This study was approved by the ethics review board of Jinling Hospital. All procedures performed in studies involving human participants were in accordance with the ethical standards of the 1964 Helsinki declaration and its later amendments or comparable ethical standards. Due to the retrospective nature of this study, patient consent was waived.

Patients were included according to the following criterion: (1) diagnosed as ischemic stroke within 14 days of onset, (2) aged ≥ 65 years old, (3) examined with a brain computed tomography or magnetic resonance imaging right before or during admission, (4) finished at least one year follow up or deceased before then. Patients were excluded if they (1) had recurrent events within the first 21days, (2) had missing fasting blood glucose and triglyceride values.

### Baseline characteristics

Demographic data, medical history, laboratory data, imaging data, and medications at discharge were all recorded. Stroke severity was assessed with the National Institute of Health Stroke Scale score (16). Stroke subtypes were classified according to the trial of ORG 10172 in Acute Stroke Treatment classification as large-artery atherosclerosis, cardio-embolism, small vessel occlusion, and others (stroke of other determined etiology and stroke of undetermined etiology) (17). Annual family incomes (1 USD = 7.18 RMB; RMB, Chinese currency) and educational years were acquired with the face-to-face questionnaire. Smoking status was classified as non-smokers, former smokers, and current smokers according to the consumption of cigarettes (18). Fasting blood samples were collected within 24 hours after admission. TyG index was calculated as ln [triglyceride (mg/dL) × fasting blood glucose (mg/dL)/2] (19).

### Follow-up and endpoints

The follow-up schedule for each patient was three, six, and twelve months and annually after the discharge. Stroke recurrence was defined as a new neurological deficit or a sudden deterioration of a previous deficit that fits the criterion of ischemic or hemorrhagic stroke, which was confirmed by clinical manifestation, neuroimaging results, death certificates, or other available data at each follow-up. The endpoint was defined as fatal or nonfatal recurrent stroke within one year of the index stroke (15).

### Statistical analysis

Continuous parameters presented as mean ± SD or median (interquartile range) were compared using Student t test or Mann-Whitney U test as appropriate. Categorized parameters presented as n (%) were compared with χ2 test or Fisher exact test. Comparison of multiple values between subgroups was compared with trend tests, one-way analysis of variance, or Kruskal-Wallis H test as appropriate. Multiple imputation method with chain equations was performed to deal with missing values.

We performed univariable Cox proportional hazards regression models to explore the association between baseline characteristics and the risk of stroke recurrence. To assess the association between TyG index and stroke recurrence, we adjusted model 1 with age and sex. Model 2 was further adjusted for hypertension, diabetes mellitus, smoking status, drinking, coronary heart disease, atrial fibrillation, antiplatelet drug, anticoagulant, statin, antihypertensive drug and hypoglycemic agent. Model 3 was adjusted for variables with the significance level of *P* < 0.1 in the univariable analysis with the back-ward selection method except for fasting blood glucose and triglyceride which were included in TyG index. We found no violations of the proportional-hazards assumption with the Schoenfeld residuals test. We also performed the competing risk analysis by accounting for the competing risk of death with the Fine and Gray method.

We explored the pattern of the association between TyG index and stroke recurrence risk with the restricted cubic spline with four knots (at 5th, 35th, 65th, and 95th percentiles) adjusted for the variables finally included in the model 3 (20). The discriminative ability of TyG index was assessed with the receiver operative characteristic and the corresponding area under the curve. Besides, we used the net improvement index (NRI) and integrated discrimination improvement (IDI) to assess the improvement of the model performance after adding TyG index into models (21).

All statistical tests were conducted with R statistical software version 4.1.0. (R Foundation, Vienna, Austria) and a two-sided *P* value < 0.05 was considered to be statistically significant.

## Results

### Baseline characteristics

**Figure 1:**
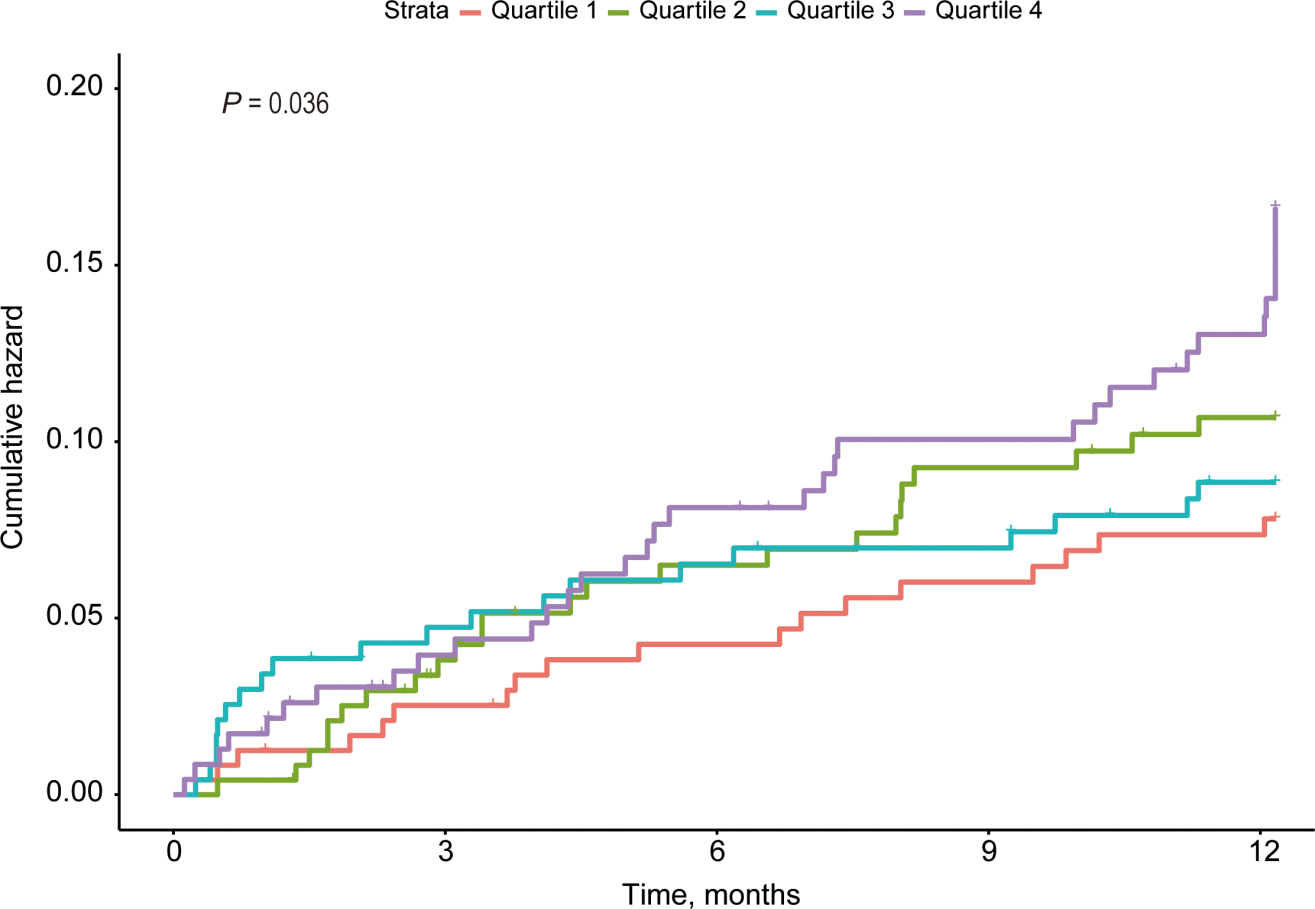
Cumulative Hazard Curves of Stroke Recurrence by TyG index.

A total of 955 elderly patients (median age, 70.0 [67.0, 75.0]; male sex, 67.2%) with ischemic stroke were included in the present study after excluding 63 patients without fasting blood glucose and triglyceride values and 37 patients without follow-up information or experienced recurrence within first 21 days. Patients with stroke recurrence had lower levels of high density lipoprotein (*P* = 0.005), higher levels of homocysteine (*P* = 0.002), fasting blood glucose (*P* < 0.001), systolic blood pressure (*P* = 0.015), and TyG index (*P* = 0.006), higher proportions of large-artery atherosclerosis and cardio-embolism (*P* = 0.029), lower annal family income (*P* = 0.039, **Table 1**. Across different quartiles of TyG index, patients with higher TyG index had higher levels of body mass index (*P* < 0.001), fasting blood glucose (*P* < 0.001) and blood urea nitrogen (*P* = 0.044), higher proportions of hypertension (*P* = 0.001), antihypertensive drug (*P* < 0.001), diabetes mellitus (*P* < 0.001), hypoglycemic agent (*P* = 0.016), dyslipidemia (*P* < 0.001), smoking (*P* = 0.001) and stroke recurrence (*P* = 0.017, **Supplementary Table 1**; *P* = 0.036, **Figure 1**), and lower proportion of male sex (*P* < 0.001, **Supplementary Table 1**).

**Table 1:** Baseline Characteristics between Patients with or without Stroke Recurrence in the Elderly.

	Without recurrence	Recurrence	
Characteristics	(N = 858)	(N = 97)	P value
Age, years	70 [67, 75]	71 [67, 77]	0.17
Male, n (%)	576 (67.1)	66 (68.0)	0.947
BMI, kg/m (2)	24.2 [22.3, 26.3]	24.8 [22.2, 26.4]	0.517
Systolic blood pressure, mmHg	143 [130, 160]	149 [134, 170]	0.015
Diastolic blood pressure, mmHg	83 [76, 90]	87 [79, 90]	0.103
Hypertension, n (%)	683 (79.6)	81 (83.5)	0.437
Diabetes mellitus, n (%)	287 (33.4)	37 (38.1)	0.417
Dyslipidemia, n (%)	201 (23.4)	28 (28.9)	0.287
Atrial fibrillation, n (%)	120 (14.0)	15 (15.5)	0.809
Coronary heart disease, n (%)	107 (12.5)	11 (11.3)	0.874
Drinking, n (%)	158 (18.4)	15 (15.5)	0.564
Smoking, n (%)			0.804
Nonsmokers	413 (48.1)	44 (45.4)	
Former smokers	112 (13.1)	12 (12.4)	
Current smokers	333 (38.8)	41 (42.3)	
NIHSS, score	3 [1, 7]	4 [2, 8]	0.086
Laboratory results			
Total cholesterol (mmol/L)	4.1 [3.5, 4.8]	4.3 [3.4, 5.0]	0.399
Triglyceride (mmol/L)	1.3 [1.0, 1.7]	1.4 [1.0, 1.9]	0.054
Low density lipoprotein (mmol/L)	2.5 [1.9, 3.0]	2.6 [1.8, 3.1]	0.677
High density lipoprotein (mmol/L)	1.0 [0.9, 1.2]	1.0 [0.8, 1.1]	0.005
Homocysteine (mmol/L)	14.7 [10.7, 19.2]	16.8 [12.6, 20.4]	0.002
Fasting blood glucose (mmol/L)	5.2 [4.6, 6.3]	5.6 [5.0, 7.7]	<0.001
Creatine (μmmol/L)	67.0 [56.0, 80.0]	70.0 [57.0, 86.0]	0.102
Blood urea nitrogen (mmol/L)	5.3 [4.5, 6.6]	5.6 [4.8, 6.8]	0.115
Uric acid, μmol/L	326 [259, 393]	331 [277, 402]	0.577
TyG	8.6 [8.3, 9.0]	8.8 [8.3, 9.3]	0.006
TOAST, n (%)			0.029
LAA	401 (46.7)	50 (51.5)	
CE	100 (11.7)	16 (16.5)	
SVD	137 (16.0)	5 (5.2)	
Others	220 (25.6)	26 (26.8)	
Education, years, n (%)			0.869
0-6	361 (42.1)	41 (42.3)	
06-9	298 (34.7)	31 (32.0)	
09-12	114 (13.3)	13 (13.4)	
>12	85 (9.9)	12 (12.4)	
Annual family Income, $, n (%)			0.039
1-1502	153 (17.8)	22 (22.7)	
1502-4506	160 (18.6)	22 (22.7)	
4506-7510	218 (25.4)	31 (32.0)	
7510-15021	241 (28.1)	19 (19.6)	
>15021	86 (10.0)	3 (3.1)	
Medication at discharge, n (%)			
Antiplatelet drug	790 (92.1)	94 (96.9)	0.13
Anticoagulant	53 (6.2)	1 (1.0)	0.065
Statin	814 (94.9)	90 (92.8)	0.529
Antihypertensive drug	462 (53.8)	53 (54.6)	0.967
Hypoglycemic agent	256 (29.8)	36 (37.1)	0.174

BMI, body mass index; CE, cardio-embolism; LAA, large-artery atherosclerosis; NIHSS, National Institute of Health Stroke Scale; SAA, small-vessel occlusion; TyG, triglyceride-glucose index.

### Association between TyG index and stroke recurrence

During one year follow-up, 97 (10.2%) patients experienced stroke recurrence, which included 79 (81.4%) ischemic and 18 (18.6%) hemorrhagic recurrent stroke, and 164 (17.1%) patients died. Univariable analysis revealed that age (hazard ratio [HR], 1.041; 95% confidence interval [CI], 1.004-1.078; *P* = 0.029), triglyceride (HR, 1.263; 95% CI, 1.133-1.408, *P <*0.001), high density lipoprotein (HR, 0.306; 95% CI, 0.129-0.724, *P* = 0.007), homocysteine (HR, 1.024; 95% CI, 1.014-1.035, *P* < 0.001), fasting blood glucose (HR, 1.133; 95% CI, 1.067-1.203, *P* < 0.001), creatine (HR, 1.003; 95% CI, 1.000-1.007, *P* = 0.041), TyG index (HR, 1.861; 95% CI, 1.400-2.475, *P* < 0.001), TOAST (small-vessel occlusion versus large-artery atherosclerosis; HR, 0.302; 95% CI, 0.121-0.758, *P* = 0.011) and annual family income (>15021 versus 1-1502; HR, 0.259; 95% CI, 0.078-0.866, *P* = 0.028; **Supplementary Table 2**).

### The ability of the TyG index to predict stroke recurrence

**Figure 2:**
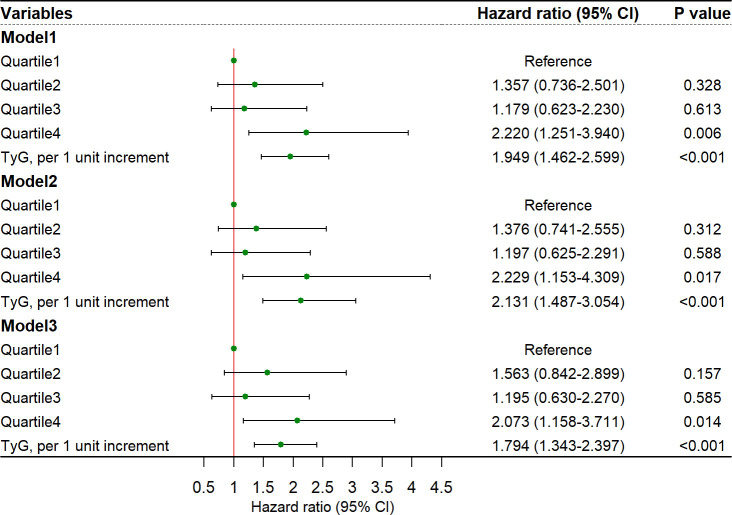
Risk of stroke recurrence in elderly patients for TyG index. CI, confidence interval, TyG, triglyceride-glucose index. Model 1: adjusted for age and sex. Model 2: adjusted for age, sex, hypertension, diabetes mellitus, smoking status, drinking, coronary heart disease, atrial fibrillation, antiplatelet drug, anticoagulant, statin, antihypertensive drug and hypoglycemic agent. Model 3: adjusted for variables included in the back-ward selection method: age, high density lipoprotein, homocysteine, annual family income, anticoagulants and stroke subtypes.

**Figure 3:**
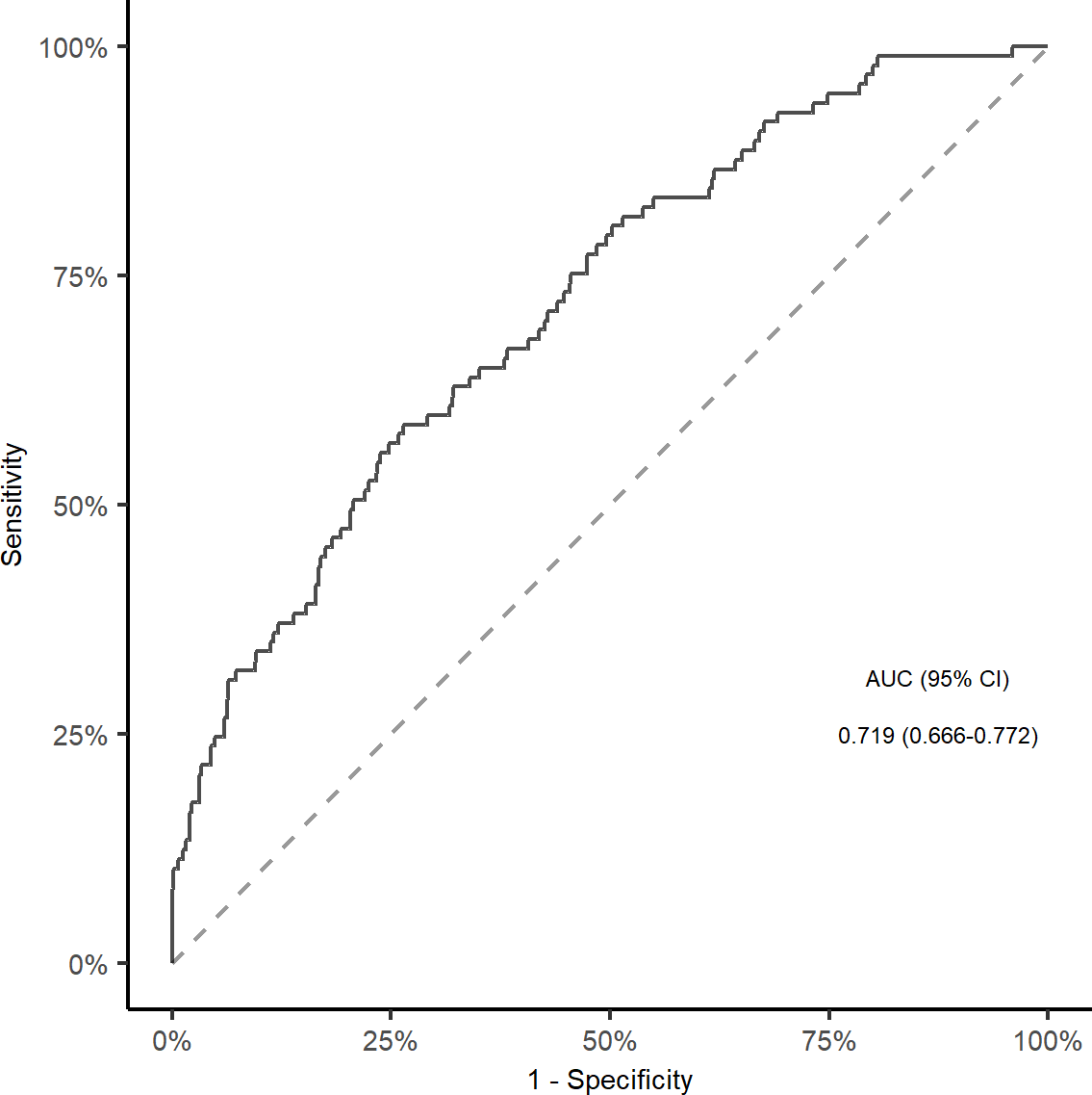
ROC curve and corresponding AUC for TyG index to predict Stroke recurrence in elderly patients. ROC, receiver operative characteristic; AUC, area under the curve; TyG, triglyceride-glucose index.

In multivariable analyses, the association between TyG index and stroke recurrence remained significant after adjusting for confounders in model 1 (quartile 4 versus quartile 1; HR, 2.220, 95% CI, 1.251-3.940; *P* = 0.006), model 2 (quartile 4 versus quartile 1; HR, 2.229, 95% CI, 1.153-4.309; *P* = 0.017), and model 3 (quartile 4 versus quartile 1; HR, 2.073, 95% CI, 1.158-3.711; *P* = 0.014; **Figure 2**). The restricted cubic spline showed an increasing trend for TyG index and stroke recurrence (*P* for non-linearity = 0.072, **Supplementary Figure 1**) after adjusting for covariables in the model 3. The area under the curve to predict stroke recurrence with TyG index was 0.719 (95% CI, 0.666-0.772, **Figure 3**). The association remained significant in the competing risk analysis accounting for the risk of death (**Supplementary Table 3**). Furthermore, adding TyG index into model 3 slightly improved the prediction of stroke recurrence (NRI (continuous), 0.142; 95% CI, 0.026-0.264, *P* = 0.020; NRI (categorical) 0.142; 95% CI, -0.032-0.260; *P* = 0.056; IDI, 0.028; 95% CI, 0.010-0.045; *P* = 0.002; **Table 2**).

**Table 2:** Reclassification Indexes for Regression Models.

Indexes	Estimate (95% CI)	*P* value
NRI (continuous)	0.142 (0.026-0.264)	0.02
NRI (categorical)	0.142 (-0.032-0.260)	0.056
IDI	0.028 (0.010-0.045)	0.002

CI, confidence interval; IDI, integrated discrimination improvement; NRI, net reclassification improvement.

## Discussion

In this study, we found that TyG index was associated with stroke recurrence in elderly patients with ischemic stroke. Besides, TyG index slightly improved the prediction for stroke recurrence. These results indicated that TyG index was a surrogate marker and might help identify patients with an increased risk of stroke recurrence in the elderly.

Stroke recurrence is common and could cause cumulative disability and cognitive impairment (22). Despite the promotion of secondary preventive drugs and the decreasing trend of the recurrence incidence, approximately 11% of patients still experience recurrence in the early stage after the index stroke (4, 23). Age is the risk factor for stroke recurrence. The incidence of stroke recurrence in the elderly varies from 7.7% to 13.1% due to different exclusion criterion (24, 25). The cumulative incidence in our study (10.2%) was similar to previous reports. Prior studies indicated that elderly patients had markedly higher risks of stroke recurrence than other age groups (23, 26). Besides, elderly patients might have less-effective treatment and poorer outcomes than younger adults (3). Accurate identification of patients at high risk of stroke recurrence in elderly patients is important to deliver effective secondary prevention and reduce the recurrence risk.

IR is a syndrome linked to metabolic disorders, such as diabetes mellitus, hypertension, obesity, and lipid disorder (27). Subsequently, these disorders are important risk factors for stroke recurrence (28). Previous studies reported that IR played an important role in the development of coronary heart disease, stroke, and cognitive dysfunction, few studies had explored the potential role of IR in the prognosis of ischemic stroke in the elderly (29). TyG index is the combination of fasting glucose and triglyceride and a reliable surrogate marker of IR. In recent years, TyG index has been suggested to assess IR in clinical practice rather than the golden standard measurement, the hyperinsulinaemic-euglycaemic clamp test, due to the convenience and reliability (19).

Prior studies showed that TyG index was associated with ischemic stroke. A community-based cohort showed that elevated levels of TyG index could independently predict ischemic stroke during an 11-year follow-up in the general population regardless of the sampling time (9). Guo et al. explored the relationship between TyG index and platelet reactivity in patients with acute ischemic stroke and found that elevated TyG index could enhance platelet reactivity. Nam et al. found that TyG index was associated with early recurrent ischemic lesions in a small sample of patients with acute ischemic stroke (30). Unfortunately, studies focusing on the elderly population were limited. A longitudinal study performed among the elderly showed that TyG index had a superior discriminative ability for the occurrence of hypertension over lipid parameters (31). The Northern Shanghai Study revealed that TyG index was associated with macro- and microvascular damage in elderly individuals (32). In our study, we found that TyG index was associated with stroke recurrence and traditional risk factors such as hypertension and diabetes mellitus in the elderly. TyG index might help identify high risk patients who might benefit from interventions for IR including weight control, physical activity and healthy diets.

The mechanism underlying the association between TyG index and stroke recurrence might be explained as follows. First, IR could affect platelet adhesion, aggregation, and activation and was associated with artery stenosis and occlusion (33, 34). Second, IR might result in chronic inflammation (35), endothelium dysfunction (36), and the formation of foam cells (37). Previous studies suggested that TyG index was related to arterial stiffness in the elderly and thus might contribute to stroke recurrence (38). Third, beyond the specific setting of diabetes, TyG index was also associated with subclinical atherosclerosis (39), coronary atherosclerosis in the general population (40), and carotid plaque stability in nondiabetic adults (41), which were important predictors of ischemic events (42). Fourth, IR might coexist with a cluster of traditional risk factors, such as hypertension, obesity, and diabetes mellitus (9), and contribute to stroke recurrence development.

Besides, we found that homocysteine, stroke subtypes, and family income were associated with stroke recurrence in the elderly. Zhang et al. performed a prospective cohort and found that elevated homocysteine can predict stroke recurrence and mortality in patients with stroke (43). Shi et al. found that homocysteine was associated with stroke recurrence in patients with large-vessel atherosclerosis (44). Large-vessel atherosclerosis is the most common subtype in the Chinese population (45). Lange et al. found that patients with atherosclerosis in the internal carotid artery, intracranial and posterior circulation had an increased risk of stroke recurrence (46). Besides, Flach et al. reported that cardio-embolic stroke also had a higher risk of recurrence (47). Socioeconomic status was also associated with stroke recurrence. Chen et al. suggested that patients with lower socioeconomic status might have less access to acute interventions and were more disobedient to the secondary prevention treatments (48). Our results were in agreement with these previous findings.

However, our study had several limitations. First, this was a retrospective analysis of a prospective database that included patients aged ≥ 65 years, which might generate sampling bias. Second, socioeconomic status information was collected by questionnaires, which might generate information bias. Third, we selected stroke recurrence within one year as the endpoint because of the higher recurrence rate within one year and the lower rate of patients without follow-up (49), however, long-term follow-up information was still warranted in the future. Forth, limited by the study design, the time-varying change of TyG index after discharge was not provided, which might provide more information. Fifth, we lacked information about transient ischemic attacks and the patterns of adherence or persistence of medication after discharge, however, we provided medication at discharge instead. Finally, although TyG index was validated to be correlated with the hyperinsulinaemic-euglycaemic clamp test, we were unable to compare the performance of the hyperinsulinaemic-euglycaemic clamp test in our study because of the retrospective nature.

In conclusion, the results of our study showed that elevated TyG index was associated with stroke recurrence in elderly patients with ischemic stroke. Further studies are warranted to assess the role of TyG index in the development of stroke recurrence in the elderly.

## Data availability statement

The raw data supporting the conclusions of this article will be made available by the authors, without undue reservation.

## Ethics statement

The studies involving human participants were reviewed and approved by the ethics review board of Jinling Hospital. Written informed consent for participation was not required for this study in accordance with the national legislation and the institutional requirements.

## Author contributions

FW, JW, and YH contributed to conception and design of the study. FW, XS, and CH organized the database. FW and XX performed the statistical analysis. FW wrote the first draft of the manuscript. SZ and JG wrote sections of the manuscript. All authors contributed to manuscript revision, read, and approved the submitted version.

## Funding

The project was supported by National Natural Science Foundation of China (NO. U20A20357, 81901248, 81870946, and 81530038), High Level Project of Medicine in Longhua, ShenZhen (HLPM201907020102), and Construction Funds of Key Medical Disciplines in Longhua District, ShenZhen (MKD202007090208).

## Conflict of interest

The authors declare that the research was conducted in the absence of any commercial or financial relationships that could be construed as a potential conflict of interest.

## Publisher’s note

All claims expressed in this article are solely those of the authors and do not necessarily represent those of their affiliated organizations, or those of the publisher, the editors and the reviewers. Any product that may be evaluated in this article, or claim that may be made by its manufacturer, is not guaranteed or endorsed by the publisher.
