# Supplementary material for: Triglyceride-glucose index and stroke recurrence in elderly patients with ischemic stroke
Source: Front Endocrinol (Lausanne). 2022 Aug 29;13:1005614. doi: 10.3389/fendo.2022.1005614 (PMC9467280; doi:10.3389/fendo.2022.1005614)
Supplement: Supplementary file 1 [file DataSheet_1.docx]

**Supplementary materials**

**Table 1. Baseline Characteristics According to Quartiles of TyG Index.**

|  | **Quartile 1** | **Quartile 2** | **Quartile 3** | **Quartile 4** |  |
| --- | --- | --- | --- | --- | --- |
| **Characteristics** | (N = 241) | (N = 242) | (N = 238) | (N = 234) | ***P* value^*^** |
| Age, years | 70 [67, 75] | 71 [67, 75] | 70 [67, 75] | 70 [66, 74] | 0.148 |
| Male, n (%) | 180 (74.7) | 167 (69.0) | 159 (66.8) | 136 (58.1) | <0.001 |
| BMI, kg/m2 | 23.3 [21.2, 25.1] | 24.2 [22.5, 26.4] | 24.5 [22.9, 26.5] | 24.8 [22.9, 27.0] | <0.001 |
| Systolic blood pressure, mmHg | 145 [132, 160] | 141 [130, 159] | 145 [131, 160] | 146 [134, 160] | 0.085 |
| Diastolic blood pressure, mmHg | 85 [79, 90] | 81 [75, 90] | 82 [75, 90] | 83 [76, 90] | 0.149 |
| Hypertension, n (%) | 171 (71.0) | 202 (83.5) | 193 (81.1) | 198 (84.6) | 0.001 |
| Diabetes mellitus, n (%) | 43 (17.8) | 50 (20.7) | 83 (34.9) | 148 (63.2) | <0.001 |
| Dyslipidemia, n (%) | 25 (10.4) | 37 (15.3) | 52 (21.8) | 115 (49.1) | <0.001 |
| Atrial fibrillation, n (%) | 39 (16.2) | 34 (14.0) | 38 (16.0) | 24 (10.3) | 0.119 |
| Coronary heart disease, n (%) | 26 (10.8) | 30 (12.4) | 30 (12.6) | 32 (13.7) | 0.353 |
| Drinking, n (%) | 45 (18.7) | 41 (16.9) | 45 (18.9) | 42 (17.9) | 0.986 |
| Smoking, n (%) |  |  |  |  | 0.001 |
| Nonsmokers | 98 (40.7) | 115 (47.5) | 115 (48.3) | 129 (55.1) |  |
| Former smokers | 46 (19.1) | 29 (12.0) | 28 (11.8) | 21 (9.0) |  |
| Current smokers | 97 (40.2) | 98 (40.5) | 95 (39.9) | 84 (35.9) |  |
| NIHSS, score | 3 [1, 7] | 3 [1, 7] | 4 [1, 8] | 3 [2, 8] | 0.377 |
| Laboratory results |  |  |  |  |  |
| Total cholesterol (mmol/L) | 3.7 [3.2, 4.3] | 4.1 [3.4, 4.7] | 4.2 [3.6, 4.8] | 4.6 [3.8, 5.5] | <0.001 |
| Triglyceride (mmol/L) | 0.8 [0.7, 1.0] | 1.2 [1.0, 1.3] | 1.5 [1.3, 1.7] | 2.0 [1.6, 2.6] | <0.001 |
| Low density lipoprotein (mmol/L) | 1.1 [0.9, 1.3] | 1.1 [0.9, 1.3] | 1.0 [0.9, 1.2] | 1.0 [0.8, 1.1] | <0.001 |
| High density lipoprotein (mmol/L) | 2.2 [1.7, 2.7] | 2.4 [1.9, 3.0] | 2.6 [2.1, 3.1] | 2.6 [2.0, 3.2] | <0.001 |
| Homocysteine (mmol/L) | 14.8 [10.9, 19.4] | 15.6 [11.2, 19.3] | 15.4 [10.9, 20.2] | 13.8 [10.7, 18.6] | 0.238 |
| Fasting blood glucose (mmol/L) | 4.6 [4.2, 5.0] | 5.0 [4.5, 5.6] | 5.5 [4.9, 6.4] | 7.3 [5.7, 9.6] | <0.001 |
| Creatine (μmmol/L) | 67.0 [56.0, 78.0] | 68.0 [57.0, 80.8] | 68.0 [56.0, 83.0] | 65.0 [54.0, 78.0] | 0.188 |
| Blood urea nitrogen (mmol/L) | 5.3 [4.4, 6.2] | 5.3 [4.6, 6.6] | 5.6 [4.6, 6.8] | 5.3 [4.6, 6.9] | 0.044 |
| Uric acid, μmol/L | 332 [260, 395] | 323 [265, 394] | 327 [254, 391] | 325 [260, 390] | 0.951 |
| TOAST, n (%) |  |  |  |  | 0.265 |
| LAA | 120 (49.8) | 110 (45.5) | 121 (50.8) | 100 (42.7) |  |
| CE | 31 (12.9) | 32 (13.2) | 31 (13.0) | 22 (9.4) |  |
| SVD | 32 (13.3) | 34 (14.0) | 34 (14.3) | 42 (17.9) |  |
| Others | 58 (24.1) | 66 (27.3) | 52 (21.8) | 70 (29.9) |  |
| Education, years, n (%) |  |  |  |  | 0.410 |
| 0-6 | 112 (46.5) | 90 (37.2) | 102 (42.9) | 98 (41.9) |  |
| 6-9 | 72 (29.9) | 89 (36.8) | 86 (36.1) | 82 (35.0) |  |
| 9-12 | 30 (12.4) | 34 (14.0) | 28 (11.8) | 35 (15.0) |  |
| >12 | 27 (11.2) | 29 (12.0) | 22 (9.2) | 19 (8.1) |  |
| Annual family Income, $, n (%) |  |  |  |  | 0.293 |
| 1-1502 | 47 (19.5) | 33 (13.6) | 46 (19.3) | 49 (20.9) |  |
| 1502-4506 | 44 (18.3) | 46 (19.0) | 44 (18.5) | 48 (20.5) |  |
| 4506-7510 | 57 (23.7) | 71 (29.3) | 56 (23.5) | 65 (27.8) |  |
| 7510-15021 | 65 (27.0) | 69 (28.5) | 67 (28.2) | 59 (25.2) |  |
| >15021 | 28 (11.6) | 23 (9.5) | 25 (10.5) | 13 (5.6) |  |
| Medication at discharge, n (%) |  |  |  |  |  |
| Antiplatelet drug | 227 (94.2) | 222 (91.7) | 217 (91.2) | 218 (93.2) | 0.624 |
| Anticoagulant | 8 (3.3) | 19 (7.9) | 15 (6.3) | 12 (5.1) | 0.554 |
| Statin | 231 (95.9) | 228 (94.2) | 224 (94.1) | 221 (94.4) | 0.505 |
| Antihypertensive drug | 121 (50.2) | 127 (52.5) | 121 (50.8) | 146 (62.4) | <0.001 |
| Hypoglycemic agent | 36 (14.9) | 49 (20.2) | 65 (27.3) | 142 (60.7) | 0.016 |
| Recurrence, n (%) | 18 (7.5) | 24 (9.9) | 20 (8.4) | 35 (15.0) | 0.017 |

Abbreviations: BMI, body mass index; CE, cardio-embolism; LAA, large-artery atherosclerosis; NIHSS, National Institute of Health Stroke Scale; SAA, small-vessel occlusion; TyG, triglyceride-glucose index.

**Table 2. Univariable Analysis of Risk factors for Stroke Recurrence.**

| **Characteristics** | **Hazard ratio (95% CI)** | ***P* value** |
| --- | --- | --- |
| Age, years | 1.041 (1.004-1.078) | 0.029 |
| Male, n (%) | 1.039 (0.678-1.592) | 0.860 |
| BMI, kg/m^2^ | 1.018 (0.957-1.082) | 0.578 |
| Systolic blood pressure, mmHg | 1.010 (0.999-1.021) | 0.086 |
| Diastolic blood pressure, mmHg | 1.013 (0.997-1.030) | 0.120 |
| Hypertension, n (%) | 1.279 (0.748-2.187) | 0.368 |
| Diabetes mellitus, n (%) | 1.208 (0.802-1.820) | 0.366 |
| Dyslipidemia, n (%) | 1.284 (0.828-1.992) | 0.265 |
| Atrial fibrillation, n (%) | 1.142 (0.659-1.980) | 0.637 |
| Coronary heart disease, n (%) | 0.895 (0.478-1.676) | 0.729 |
| Drinking, n (%) | 0.813 (0.469-1.410) | 0.461 |
| Smoking, n (%) |  |  |
| Nonsmokers | Reference |  |
| Former smokers | 0.970 (0.512-1.836) | 0.925 |
| Current smokers | 1.155 (0.755-1.767) | 0.507 |
| NIHSS, score | 1.022 (0.992-1.053) | 0.149 |
| Laboratory results |  |  |
| Total cholesterol (mmol/L) | 1.057 (0.877-1.275) | 0.561 |
| Triglyceride (mmol/L) | 1.263 (1.133-1.408) | <0.001 |
| Low density lipoprotein (mmol/L) | 1.032 (0.823-1.293) | 0.786 |
| High density lipoprotein (mmol/L) | 0.306 (0.129-0.724) | 0.007 |
| Homocysteine (mmol/L) | 1.024 (1.014-1.035) | <0.001 |
| Fasting blood glucose (mmol/L) | 1.133 (1.067-1.203) | <0.001 |
| Creatine (μmmol/L) | 1.003 (1.000-1.007) | 0.041 |
| Blood urea nitrogen (mmol/L) | 1.062 (0.989-1.141) | 0.096 |
| Uric acid, μmol/L | 1.000 (0.998-1.002) | 0.720 |
| TyG | 1.861 (1.400-2.475) | <0.001 |
| TOAST, n (%) |  |  |
| LAA | Reference |  |
| CE | 1.294 (0.737-2.273) | 0.369 |
| SVD | 0.302 (0.121-0.758) | 0.011 |
| Others | 0.938 (0.584-1.506) | 0.790 |
| Education, years, n (%) |  |  |
| 0-6 | Reference |  |
| 6-9 | 0.926 (0.581-1.477) | 0.747 |
| 9-12 | 1.018 (0.545-1.899) | 0.956 |
| >12 | 1.242 (0.653-2.363) | 0.510 |
| Annual family Income, $, n (%) |  |  |
| 1-1502 | Reference |  |
| 1502-4506 | 0.964 (0.534-1.741) | 0.903 |
| 4506-7510 | 0.992 (0.574-1.713) | 0.977 |
| 7510-15021 | 0.564 (0.305-1.041) | 0.067 |
| >15021 | 0.259 (0.078-0.866) | 0.028 |
| Medication at discharge, n (%) |  |  |
| Antiplatelet drug | 2.429 (0.769-7.667) | 0.130 |
| Anticoagulant | 0.174 (0.024-1.244) | 0.081 |
| Statin | 0.617 (0.286-1.333) | 0.219 |
| Antihypertensive drug | 1.019 (0.683-1.519) | 0.927 |
| Hypoglycemic agent | 1.361 (0.901-2.054) | 0.143 |

Abbreviations: BMI, body mass index; CE, cardio-embolism; CI, confidence interval; LAA, large-artery atherosclerosis; NIHSS, National Institute of Health Stroke Scale; SAA, small-vessel occlusion; TyG, triglyceride-glucose index.

**Table 3. Competing Risk Analysis of TyG index and Stroke Recurrence.**

|  | **Hazard ratio (95% CI)** | ***P* value** |
| --- | --- | --- |
| Model 1 | 1.970 (1.414-2.750) | <0.001 |
| Model 2 | 1.996 (1.323-3.010) | 0.001 |
| Model 3 | 1.822 (1.300-2.550) | <0.001 |

Abbreviations: CI, confidence interval, TyG, triglyceride-glucose index.

Model 1: adjusted for age and sex.

Model 2: adjusted for age, sex, hypertension, diabetes mellitus, smoking status, drinking, coronary heart disease, atrial fibrillation, antiplatelet drug, anticoagulant, statin, antihypertensive drug and hypoglycemic agent.

Model 3: adjusted for variables included in the back-ward selection method: age, high density lipoprotein, homocysteine, annual family income, anticoagulants and stroke subtypes.

**Figure 1. Adjusted hazard ratio of stroke recurrence in elderly patients by TyG index.**


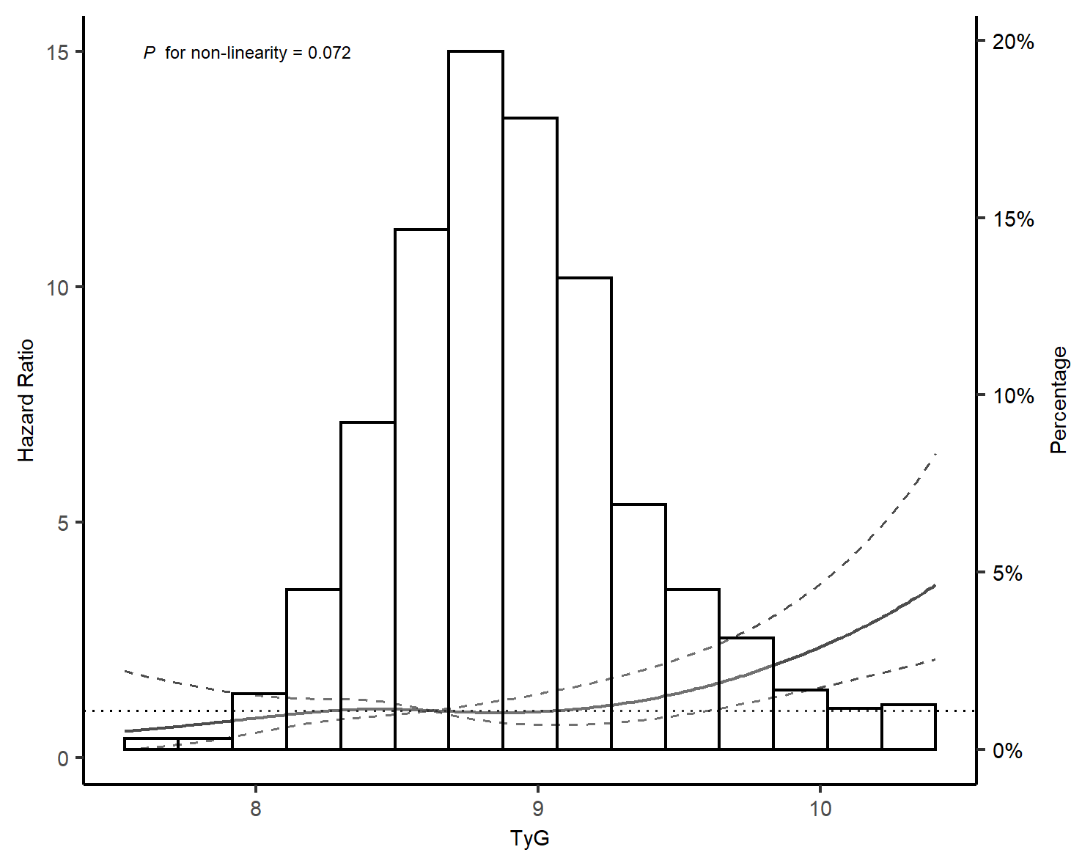


Abbreviations: Hazard ratio was compared with a median TyG index of 8.6 in model 3. TyG, triglyceride-glucose index.
